# Diencephalic organoids – A key to unraveling development, connectivity, and pathology of the human diencephalon

**DOI:** 10.3389/fncel.2023.1308479

**Published:** 2023-12-07

**Authors:** Ferdi Ridvan Kiral, Museog Choe, In-Hyun Park

**Affiliations:** Interdepartmental Neuroscience Program, Department of Genetics, Yale Stem Cell Center, Yale Child Study Center, Wu Tsai Institute, Yale School of Medicine, New Haven, CT, United States

**Keywords:** diencephalon, thalamus, brain organoids, assembloids, neurodevelopmental disorders

## Abstract

The diencephalon, an integral component of the forebrain, governs a spectrum of crucial functions, ranging from sensory processing to emotional regulation. Yet, unraveling its unique development, intricate connectivity, and its role in neurodevelopmental disorders has long been hampered by the scarcity of human brain tissue and ethical constraints. Recent advancements in stem cell technology, particularly the emergence of brain organoids, have heralded a new era in neuroscience research. Although most brain organoid methodologies have hitherto concentrated on directing stem cells toward telencephalic fates, novel techniques now permit the generation of region-specific brain organoids that faithfully replicate precise diencephalic identities. These models mirror the complexity of the human diencephalon, providing unprecedented opportunities for investigating diencephalic development, functionality, connectivity, and pathophysiology *in vitro*. This review summarizes the development, function, and connectivity of diencephalic structures and touches upon developmental brain disorders linked to diencephalic abnormalities. Furthermore, it presents current diencephalic organoid models and their applications in unraveling the intricacies of diencephalic development, function, and pathology in humans. Lastly, it highlights thalamocortical assembloid models, adept at capturing human-specific aspects of thalamocortical connections, along with their relevance in neurodevelopmental disorders.

## 1 Introduction

The study of the human brain with its intricate complexities has always been at the front line of scientific exploration. However, understanding the development, function, and pathology of the human brain presents a formidable challenge, primarily due to the difficulty of acquiring human tissue for research and ethical considerations. Recent strides in stem cell technology have addressed these challenges with a groundbreaking solution: brain organoids, three-dimensional (3D) models of the human brain grown *in vitro* from pluripotent stem cells (Lancaster et al., 2013; Di Lullo and Kriegstein, 2017; Velasco et al., 2020). As a new research model, brain organoids swiftly carved out a distinct niche, offering unique opportunities to study the complex aspects of human brain development, structure, function, and disease that are not accessible in animal models.

Two approaches have been utilized in generating brain organoids: guided and unguided methods. Guided methods, which are used to generate organoids collectively known as region-specific brain organoids, encompass techniques of directed differentiation involving intentional manipulation of the cellular environment. These approaches entail adding specific growth factors, signaling molecules, or genetic modifications to steer cells along a predetermined developmental trajectory. Brain organoids generated by guided methods faithfully recapitulate both the developmental process and functions of a specific brain region. In contrast, unguided methods rely on spontaneous differentiation, allowing organoids to develop without direct manipulation. Cultured within an environment that supports self-organization, these organoids exhibit diverse cell types and structures akin to the human brain during embryonic development. However, although brain organoids generated with unguided methods capture the natural complexity and cellular diversity of the developing human brain, they lack the precise developmental control necessary to study specific brain regions or cell types. Conversely, guided methods enable targeted investigation of the development and function of a specific brain region (Qian et al., 2019; Mayhew and Singhania, 2022; Susaimanickam et al., 2022).

Brain development begins early in embryogenesis and involves tightly coordinated signaling cascades and cellular dynamics. Upon gastrulation, the neural plate emerges and undergoes specialization into distinct progenitor domains, each destined to contribute to the major brain regions such as telencephalon, diencephalon, mesencephalon, metencephalon, and myelencephalon (Stiles and Jernigan, 2010). The shaping of these structures relies on organizers releasing morphogens in a precise spatiotemporal manner to pattern the neural tube along the dorsal-ventral and anterior-posterior axes (Wilson and Maden, 2005; Sansom and Livesey, 2009; Kiecker and Lumsden, 2012). In recent years, several methods have emerged, enabling the manipulation of human pluripotent stem cells to generate region-specific brain organoids that recapitulate these developmental processes *in vitro*. These organoids encompass various types, including cortical and hippocampal organoids representing telencephalic fate, midbrain organoids derived from mesencephalic fate, and thalamic organoids emulating the diencephalic fate, among others (Jo et al., 2016; Xiang et al., 2017, 2019; Cakir et al., 2019; Qian et al., 2020; Kim et al., 2023b; Kiral et al., 2023). In this mini-review, we will summarize the development, function, and connectivity of the main structures in the diencephalon in both healthy and pathological conditions. Additionally, we will discuss the current models of diencephalic organoids and their use in understanding the development, connectivity, and pathology of the human diencephalon.

## 2 The diencephalon and neurodevelopmental disorders

### 2.1 Development and functions of the diencephalic structures

During early embryogenesis, the diencephalon originates from the prosencephalon, a segment of the developing neural tube that forms the forebrain. Following gastrulation, the prosencephalon undergoes a series of morphological transformations, eventually splitting into the telencephalon anteriorly and the diencephalon posteriorly. The telencephalon contributes to the formation of the cerebral cortex, hippocampus, and basal ganglia, whereas the diencephalon gives rise to the structures, including the pretectum, thalamus, epithalamus, and prethalamus (Jessell and Sanes, 2000; Colas and Schoenwolf, 2001). Over the past century, embryologists have proposed two contrasting models to elucidate the fundamental principles of forebrain development, both sharing a common feature: the concept that development occurs through neighboring modules or independent units. On the one hand, the columnar model, initially introduced by Herrick in 1910 and later expanded upon by Alvarez-Bolado et al. in 1995, posits that the forebrain’s core components are organized as longitudinal columns along the dorsal-ventral axis. According to this model, the diencephalon is situated between the telencephalon in the rostral direction and the mesencephalon (midbrain) in the caudal direction, and it is further divided into four segments, progressing from dorsal to ventral: the epithalamus, thalamus, ventral thalamus, and hypothalamus (Herrick, 1910; Alvarez-Bolado et al., 1995). On the other hand, the prosomeric model, originally proposed by Bergquist in 1932 and later refined by Puelles and Rubenstein in 2003, offers an alternative perspective. This model interprets the division of the diencephalon into functional units based on distinct anatomical regions and gene expression patterns, establishing itself as a widely accepted framework for comprehending forebrain development (Bergquist, 1932; Puelles and Rubenstein, 2003). The prosomeric model places emphasis on the anterior-posterior patterning and segmentation of the diencephalon, dividing it into three primary prosomeres. Prosomere 1 (p1) matures into the pretectum that is primarily responsible for controlling functions related to eye movement and visual coordination (Gamlin, 2006). Prosomere 2 (p2) develops dorsally into the epithalamus, housing structures like the habenula and pineal gland, and ventrally to the thalamus. The habenula connects to the midbrain and plays vital roles in reward signaling, whereas the pineal gland is specialized as a neuroendocrine organ producing the hormone melatonin and participates in daily and seasonal circadian rhythm regulation (Klein, 2004; Hikosaka, 2010; Sapede and Cau, 2013). The thalamus, with a diverse set of functionally distinct nuclei, serves as the main relay station for sensory information from peripheral sensory organs to various cortical areas (Lopez-Bendito and Molnar, 2003; Nakagawa, 2019). Lastly, prosomere 3 (p3) gives rise to the prethalamus, with the most prominent structure called the thalamic reticular nucleus (TRN). The TRN, a sheet of inhibitory GABAergic neurons situated between the thalamus and cortex, modulates thalamic and cortical inputs, regulating attention, arousal, sensory and motor integration, and network oscillations (McAlonan et al., 2006; Halassa et al., 2011; Marlinski et al., 2012). In this review, we will adhere to the prosomeric model, which delineates the hypothalamus as distinct from the diencephalon. Therefore, discussions related to the hypothalamus and its organoids will not be featured in this review (Qian et al., 2016; Huang et al., 2021).

**FIGURE 1:**
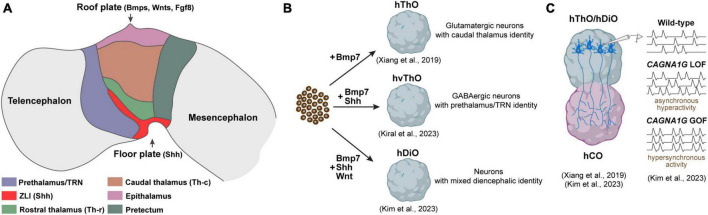
Components of the developing diencephalon and corresponding organoid models. **(A)** Diagram illustrating the primary structures within the developing diencephalon and signaling molecules released from the roof and floor plate. ZLI, Zona limitans intrathalamica. **(B)** Diencephalic organoid models generated to date. hThO, human thalamic organoid; hvThO, human ventral thalamic organoid; hDiO, human diencephalic organoid. **(C)** Assembloids to model thalamacortical connectivity. hCO, human cortical organoids; LOF, loss-of-function; GOF, gain-of-function.

According to the prosomeric model, the diencephalon undergoes division through dorso-ventral and anterior-posterior patterning, creating discrete progenitor domains. This partitioning is orchestrated by an interplay of morphogen gradients and signaling molecules that collaboratively establish unique gene expression patterns and the subsequent fate of the cells within these specific territories. Sonic Hedgehog (Shh) stands out as the key signaling molecule recognized for its role in generating diverse identities within the diencephalon. Shh is not solely produced in the ventral part, the floor plate of the diencephalon, but is also released from a transverse band that emerges between the thalamus (p2) and prethalamus (p3), known as the zona limitans intrathalamica (ZLI) (Kiecker and Lumsden, 2004). Graded Shh signaling originating from both the floor plate and the ZLI plays a pivotal role in shaping distinct domains within p2. In regions proximal to the sources of Shh, a smaller area is specified as the rostral thalamus (Th-r), while a larger region, exposed to lower Shh levels, is specified as the caudal thalamus (Th-c). Likewise, the p3 region receives high levels of Shh, particularly defining the TRN (Figure 1A). Progenitor cells exposed to high Shh levels in the prospective Th-r and TRN subsequently differentiate into GABAergic inhibitory neurons, while those in the prospective Th-c primarily differentiate into excitatory glutamatergic neurons. This initial determination of cell fate later influences the connectivity of inhibitory and excitatory nuclei. Excitatory neurons in the Th-c project to various cortical regions, whereas inhibitory neurons in the Th-r and TRN establish connections with the excitatory thalamic nuclei but not with the cortex (Hashimoto-Torii et al., 2003; Vue et al., 2009; Jeong et al., 2011). A recent analysis of single-cell transcriptomics of the human thalamus revealed a notable increase in both the quantity and diversity of GABAergic neurons. These neurons originate from ganglionic eminences and migrate to the thalamus during the second trimester of the development. Further comparative studies are needed to determine whether these GABAergic neurons are specific to humans and how they contribute to the functional organization of thalamic nuclei and their connectivity to the cortex (Kim et al., 2023a).

The dorsal-most part of the developing diencephalon, known as the roof plate, expresses the members of TGF-β and FGF superfamilies, including Bmp7, Bmp4, Wnt family members, and Fgf8 (Dickinson and McMahon, 1992; Crossley and Martin, 1995; Shimamura and Rubenstein, 1997; Lee and Jessell, 1999; Hagemann and Scholpp, 2012). In mice, the absence of Wnt/β-catenin signaling in the developing diencephalon leads to ectopic induction of prethalamic (Dlx2, Dlx5, Dlx6, and Islet1) and Th-r markers (Helt, Gata2, and Gata3) in the presumptive Th-c region suggesting that it is required for the emergence and maintenance of the Th-c thalamic identity (Bluske et al., 2009). Likewise, the downregulation of Fgf8 results in a decrease in Gbx2 expression within the Th-c region, which is likely to impact the differentiation of glutamatergic neurons and their connections to the cortex. Intriguingly, this downregulation also leads to the near absence of epithalamic nuclei, including the habenula and pineal gland (Martinez-Ferre and Martinez, 2009; Schmidt and Pasterkamp, 2017). In short, the dynamic interplay of these signaling pathways not only shapes the initial patterning of diencephalic structures but also lays the foundation for its multifaceted functions, from sensory processing to circadian rhythm regulation.

### 2.2 Diencephalic abnormalities in neurodevelopmental disorders

The thalamus is the most prominent and well-characterized part of the diencephalon. In development, the thalamus establishes connections with multiple cortical areas involved in key functions like processing sensory inputs, controlling sleep cycles, and forming memories. This expansive thalamocortical connectivity allows the thalamus to relay and modulate information flow between subcortical structures and the cortex. Given its critical roles, abnormalities in the development of thalamic nuclei and their cortical projections can lead to neurodevelopmental and psychiatric disorders. Among these, conditions such as schizophrenia (SCZ), autism spectrum disorders (ASD), and bipolar disorder (BP) have seen growing associations with developmental and functional irregularities in thalamocortical networks, which have been correlated with clinical outcomes (Chen et al., 2019; Zhang et al., 2021; Hwang et al., 2022). Numerous studies have reported reduced thalamic volume in individuals with SCZ. Post-mortem examinations have revealed not only a reduction in gray matter volume within different thalamic nuclei but also diminished fiber density and myelination in the white matter pathways connecting these thalamic nuclei to the prefrontal cortex. These findings collectively suggest impaired connectivity between the thalamus and cortex in SCZ patients (Danos et al., 2003; Kito et al., 2009; Chen et al., 2019; Zhang et al., 2021). Intriguingly, SCZ also seems to be linked to disturbances in TRN function. One of the critical roles of the TRN is to generate and modulate sleep spindles within the thalamocortical network, a process that shows reduced activity in individuals with SCZ (Ferrarelli et al., 2007; Manoach et al., 2016; Thankachan et al., 2019). While many studies have focused on schizophrenia, emerging research using neuroimaging and genomics has found shared thalamocortical circuit abnormalities across a range of psychiatric disorders. Functional magnetic resonance imaging (fMRI) and genetic analysis have revealed common disruptions in communications between the thalamus and cortex in patients with diseases such as BP, major depression, and attention-deficit hyperactivity disorders (ADHD) (Nair et al., 2013; Tu et al., 2019; Elvsashagen et al., 2021). These findings suggest that examining shared thalamic connectivity defects could provide insights into mechanisms underlying a variety of neuropsychiatric illnesses.

The dorsal part of the diencephalon harbors the epithalamic structures, notably the habenula and pineal gland. The habenula receives input and integrates information from limbic structures and the basal ganglia and, in turn, sends outputs to various midbrain structures where dopamine (DA) and serotonin (5-HT) neurons are located (Hikosaka et al., 2008; Boulos et al., 2017). Given its connections, the habenula plays a significant role in diverse physiological processes and social behaviors, including reward processing, regulation of circadian rhythms, and decision-making (Nuno-Perez et al., 2021; Lalive et al., 2022; Salaberry and Mendoza, 2022). Eventually, dysregulation of the habenula is linked to several psychiatric disorders (Germann et al., 2021; Murru et al., 2021; Sonkusare et al., 2022). While mood disorders like major depressive disorder (MDD) typically emerge during adulthood, they have displayed a consistent association with increased neuronal activity within the habenula (Browne et al., 2018; Aizawa and Zhu, 2019; Gold and Kadriu, 2019). Both pharmacological inhibition and electrical stimulation of the habenula have shown the capacity to mitigate MDD-like behaviors in animal models (Meng et al., 2011; Winter et al., 2011; Tchenio et al., 2017). In a recent study, deep-brain stimulation of the habenula has exhibited promise in alleviating symptoms among individuals with MDD (Wang et al., 2020). Emerging findings also point to the potential involvement of the pineal gland, the other epithalamic structure, in ASD. This connection was initially identified when individuals with ASD exhibited notably low levels of serum melatonin (Sumida et al., 1996). Subsequent research has indicated the beneficial impact of administering external melatonin in alleviating symptoms associated with ASD, including sleep disturbances, anxiety, as well as deficits in learning and spatial memory (Baydas et al., 2005; Olcese et al., 2009; Tian et al., 2014). In summary, abnormalities in the development and function of diverse diencephalic structures could potentially play a role in the etiology of several neurodevelopmental disorders. Ongoing and future research will provide deeper insights into the connections between diencephalic dysfunction and these disorders.

## 3 Diencephalic organoids and neurodevelopmental disorders

Brain organoids derived from pluripotent stem cells have emerged as valuable tools for investigating human brain development and disorders. Nevertheless, the majority of research efforts, until recently, have focused on creating organoids resembling the telencephalon. This focus largely emanated from the telencephalon’s direct implication in numerous brain disorders, while the precise emulation of other forebrain regions, notably the diencephalon, remained relatively unexplored until recent advancements. The pioneering work to generate organoids resembling the diencephalon was spearheaded by the Sasai group. Their approach involved exposing mouse embryonic stem cells (mESCs) to low concentrations of caudalization factor insulin and an inhibitor for MAPK/ERK kinase, which effectively enhanced the expression of early diencephalic markers, specifically Otx2 and Pax6. Moreover, they found that BMP7, which is expressed in thalamic primordium, promoted the emergence of thalamic progenitors expressing Tcf7l2, Gbx2, and Olig3. Notably, when these diencephalic organoids were transplanted into subcortical regions of mice, the thalamic neurons within them displayed a propensity to extend their axons to cortical layers, closely mirroring their behavior in the *in vivo* context (Shiraishi et al., 2017). Building upon this foundation, Xiang et al. (2019) applied a similar methodology using human embryonic stem cells (hESCs), successfully generating brain organoids resembling the human thalamus (Figure 1B). Their single-cell RNA sequencing analysis revealed the formation of distinct thalamic lineages marked by the expression of thalamic transcription factors Tcf7l2, Pax6, and Gbx2 (Xiang et al., 2019). A shared feature between these studies was the nearly uniform development of glutamatergic, excitatory thalamic neurons inside the organoids, represented by the expression of vGLUT2. This suggests that both methods effectively guide embryonic stem cells toward a caudal thalamic fate characterized by the emergence of excitatory neurons projecting to the cortex (Shiraishi et al., 2017; Xiang et al., 2019).

While the caudal thalamus is primarily composed of excitatory neurons projecting to various cortical areas, the rostral thalamus and prethalamus are predominantly populated by GABAergic inhibitory neurons. These inhibitory neurons establish connections with the caudal thalamic region but do not extend their projections to the cortex (Ferrarelli and Tononi, 2011). In an effort to expand the toolbox for diencephalic organoids, our group recently developed a method to generate ventralized thalamic organoids (vThOs) exploiting SHH treatment on an already established thalamic organoid generation protocol (Xiang et al., 2019; Kiral et al., 2023). We discovered that SHH treatment between days 14 and 22 in culture particularly induced the expression of prethalamic markers, including Dlx2, Lhx1, and Lhx5. In contrast to previously generated thalamic organoids, vThOs predominantly comprise GABAergic inhibitory neurons. These neurons express canonical TRN markers such as Spp1 and Ecel1, a nucleus derived from the prethalamus. Intriguingly, transplantation experiments unveiled distinct connectivity patterns: ESC-derived SPP1- thalamic neurons projected to the cortex, while SPP1 + TRN neurons established local connections within the thalamus, mirroring the connectivity preferences observed in *in vivo* TRN neurons (Ferrarelli and Tononi, 2011). Due to its modulatory role in thalamocortical communication, TRN dysfunction has been linked to neurodevelopmental disorders, including autism and schizophrenia (Halassa et al., 2014; Thankachan et al., 2019). To test whether vThOs could serve as a model for studying TRN-related disorders, we perturbed two TRN-enriched disease risk genes, PTCHD1 and ERBB4 (Ahrens et al., 2015; Wells et al., 2016). Although loss-of-function of these genes did not interfere with GABAergic neural differentiation and TRN specification in vThOs, they led to a significant decrease in neural activity. These findings align with similar observations made in animal models and underscore the value of vThOs as a reliable *in vitro* model for probing human TRN development and function under both healthy and pathological conditions.

Reciprocal connections between the caudal thalamus and cortex are integral to processes such as sensory and motor function, attention, and arousal (Lopez-Bendito and Molnar, 2003). While extensive research has been conducted on the development and function of thalamocortical (TC) and corticothalamic (CT) connections in rodent models, exploring uniquely human aspects of these connections has only become achievable recently. This progress has been made possible through the fusion of human thalamic and cortical organoids, collectively termed “assembloids.” Xiang et al. (2019) were the first to establish an *in vitro* thalamocortical connectivity model by fusing thalamic and cortical organoids. Upon fusion, thalamic axons preferentially projected to putative cortical plate-like regions where newly born cortical neurons are located, resembling the TC targeting in developing brain *in vivo*. Intriguingly, once fused with cortical organoids, thalamic neurons demonstrated the presence of synchronized neural activity, suggesting network-level connections in this assembloid model (Xiang et al., 2019) (Figure 1B). Recently, Kim et al. (2023b) utilized thalamo-cortical assembloids to model developmental defects linked to the voltage-gated calcium channel Cav3.1, encoded by CACNA1G, which is enriched in the thalamus (Figure 1C). They demonstrated that a gain-of-function CACNA1G variant, previously tied to absence seizures in animal epilepsy models, led to hypersynchronous firing of both thalamic and cortical neurons in the assembloid system (Kunii et al., 2020; Kim et al., 2023b). In contrast, loss of CACNA1G, associated with schizophrenia, promoted overgrown thalamic projections and increased asynchronous spontaneous activity (Singh et al., 2022; Kim et al., 2023b). Overall, these pioneering studies highlight the potential of thalamo-cortical assembloids to uncover human-specific features of thalamocortical development and provide platforms to elucidate how thalamus-specific neurodevelopmental insults can disturb thalamo-cortical wiring and dynamics underlying neurological disorders.

## 4 Conclusion and future directions

Brain organoid systems have rapidly advanced capabilities to model distinct regions and cell types of the developing human brain. Recent progress in diencephalic organoids provides exciting new platforms to investigate spatiotemporal organization and circuit wiring of human diencephalic structures in physiological and disease states. Still, continued efforts to generate more intricate diencephalic structures and physiologically relevant assembloid models are needed. Similar to our work to generate diencephalic organoids with thalamic reticular nucleus identity, future works may exploit a combination of various signaling molecules to generate *in vitro* models for other diencephalic structures such as pretectum and epithalamus. Additionally, the current thalamo-cortical assembloid models could be improved by incorporating other essential components of the thalamocortical circuit, such as GABAergic neurons of the thalamic reticular nucleus and corridor cells of the lateral ganglionic eminence. Given that the thalamus receives sensory input from various sensory organs, incorporating sensory cells into assembloid models could further elevate their physiological relevance. Finally, similar to cortical organoids that fail to demonstrate arealization, thalamic organoids lack the formation of nuclei that are known to connect different cortical areas. Future studies need to explore methods to generate such organoids encompassing distinct nuclei, which is imperative to model nuclei-specific connectivity between the thalamus and cortex *in vitro*. In summary, diencephalic organoids represent a rapidly evolving field that offers unique opportunities to deepen our understanding of human diencephalon development, function, and dysfunction. Continued refinement of diencephalic organoid systems will expand our capabilities for investigating the origins and potential therapies for a range of neurodevelopmental disorders.

## Author contributions

FK: Conceptualization, Writing – original draft, Writing – review & editing. MC: Writing – review & editing. I-HP: Conceptualization, Funding acquisition, Resources, Writing – review & editing.
